# The Formation of Perovskite during the Combustion of an Energy-Rich Glycine–Nitrate Precursor

**DOI:** 10.3390/ma13225091

**Published:** 2020-11-11

**Authors:** Oksana V. Komova, Svetlana A. Mukha, Anna M. Ozerova, Galina V. Odegova, Valentina I. Simagina, Olga A. Bulavchenko, Arcady V. Ishchenko, Olga V. Netskina

**Affiliations:** Boreskov Institute of Catalysis, Pr. Akademika Lavrentieva 5, 630090 Novosibirsk, Russia; komova@catalysis.ru (O.V.K.); msa@catalysis.ru (S.A.M.); ozerova@catalysis.ru (A.M.O.); odegova@catalysis.ru (G.V.O.); simagina@catalysis.ru (V.I.S.); isizy@catalysis.ru (O.A.B.); arcady.ishchenko@gmail.com (A.V.I.)

**Keywords:** perovskite, solution combustion synthesis, volume combustion synthesis, self-propagating high-temperature synthesis, glycine

## Abstract

The effect of different regimes of combustion of glycine–nitrate precursors on the formation of perovskite phases (LaMnO_3_ and LaCrO_3_) without additional heat treatment was studied. The following three combustion regimes were compared: the traditional solution combustion synthesis (SCS), volume combustion synthesis (VCS) using a powdered precursor, and self-propagating high-temperature synthesis (SHS) using a precursor pellet. The products of combustion were studied using a series of physicochemical methods (attenuated total reflection infrared spectroscopy (ATR FTIR), X-ray diffraction (XRD), high-resolution transmission electron microscopy (HRTEM), and thermal analysis). SHS was found to be the most productive regime for the formation of perovskite because of its ability to develop high temperatures in the reaction zone, which led to a reduced content of the thermally stable lanthanum carbonate impurities and to an increased yield and crystallite size of the perovskite phase. The reasons for the better crystallinity and purity of LaCrO_3_ as compared with LaMnO_3_ is also discussed, namely the low temperatures of the onset of the thermolysis, the fast rate of combustion, and the favorable thermodynamics for the achievement of high temperatures in the reaction zone.

## 1. Introduction

The utilization of the heat of chemical reactions in the synthesis of various inorganic compounds was first proposed by a group of Soviet scientists under the guidance of Academician A.G. Merzhanov as far back as the 1960s [1,2]. Such processes were called self-propagating high-temperature synthesis (SHS). The first intensive studies in this area were performed for systems that burn without the evolution of gases, for example, in the synthesis of borides, carbides, and silicides of different metals. Later, these studies were extended to include the processes of combustion taking place with the evolution of gaseous products and the formation of a solid combustion product. Such processes were also called SHS [3,4,5,6,7,8].

Today, this method has also found wide application in the synthesis of complex oxides, including perovskites. Interest in perovskites was determined by the versatility of their applications (electrodes, gas detectors, membranes, etc.) [9,10,11,12,13,14,15,16,17,18,19,20]. Among them, catalysis has been a significant area where they find application both as the support materials and the active components in the gas-phase and liquid-phase reactions of complete and partial oxidation, hydrogenation, organic synthesis, photochemical conversions, and others [8,17,21,22,23].

The methods for producing complex oxides by combustion of organometallic precursors are rather similar [3,7,8,24,25,26]. First, a solution of metal nitrates and an organic material (amino acids, urea, citric acid, ethylene glycol, saccharose, etc.) is prepared. Then, the solution is heated under different conditions, which leads to its evaporation to a viscous gel-like precursor and ignition. This method is designated as solution combustion synthesis (SCS).

The SCS method is simple and versatile. It allows the preparation of oxide compounds with different compositions and structures. It also ensures a uniform distribution of the metal cations throughout the gel-like precursor as a result of their coordination with the molecules of organic ligand, a fast burning (within seconds), and a high degree of dispersion of the solid product as a result of the vigorous evolution of gases during the combustion. With the properly chosen reaction parameters, a pure and well-crystallized phase of the complex oxide can be obtained during the stage of combustion, which excludes the necessity of its further heat treatment [27,28]. However, even if the stage of heat treatment is still necessary to obtain a product of high purity, it can be carried out under milder conditions as compared with the traditional ceramic method.

One of the SCS’s shortcomings is the frequently observed explosion-like character of the burning. At the uniform heating of the entire gel-like precursor, all its mass starts to burn simultaneously. It is accompanied by an outburst of flame, gases, and combustion products. This regime of combustion is difficult to control, and in the literature it is referred to as volume combustion synthesis (VCS) [3,4].

It has been shown that the nature of the organic component [3,7,8,12,24,29,30] and the cations of the metals [31], their interaction [24,32], and the gas atmosphere [3,7] are important factors determining the course of the combustion process and the characteristics of the product. The fuel/oxidant ratio in the precursor is also an important parameter [7,11,27,29,33,34,35]. As a rule, this ratio is expressed as the elemental stoichiometric coefficient, ϕ [24]. At ϕ = 1, no oxygen is required to completely oxidize the fuel in the reaction mixture. At a shortage of the fuel (ϕ < 1), the mixture is rich in the oxidant and oxygen is one of the reaction products. At an excess of the fuel (ϕ > 1), the mixture experiences a shortage of the oxidant and therefore a supply of oxygen is necessary.

Published thermodynamic calculations show that the evaporation of water from the precursor and the heating of water vapors lead to substantial losses of heat and a decrease in temperature in the combustion zone [3,4,31,32,36]. In spite of this, only a few studies have been published where the precursors were pre-dried prior to their combustion [3,10,37], and a couple of literature sources were found devoted to the study of the effect of combustion regimes on the characteristics of the end product [3,4,29,33,38,39,40]. For combustion proceeding with the evolution of gases, practically no attempt has been made to study the potential of the more controllable SHS combustion regime, where the combustion of a pre-dried organometallic precursor (placed in a boat or pressed as a pellet) was initiated by a short local heating followed by self-propagation of the combustion wave through the volume of the precursor [10,37,39,40].

In this work, results are presented on the effect of different combustion regimes of glycine–nitrate precursors (ϕ = 1.8) and the nature of transition metal cations (Cr, Mn) on the purity and characteristics of the perovskites (LaCrO_3_, LaMnO_3_) forming during the stage of combustion without the additional calcination. The following three combustion regimes were compared: the traditional SCS, VCS using a dried and powdered gel-like precursor, and SHS using a pellet of dried precursor.

## 2. Materials and Methods

### 2.1. Preparation of Glycine–Nitrate Precursors

The following reagents were used: analytically pure Cr(NO_3_)_3_·9H_2_O (Reachem, Moscow, Russia); analytically pure Mn(NO_3_)_2_∙4H_2_O (Reachem, Moscow, Russia); La(NO_3_)_3_∙6H_2_O–99 wt% (Sigma-aldrich, St. Louis, MO, USA); pure grade glycine C_2_H_5_O_2_N (Reachem, Moscow, Russia); and special purity HNO_3_ (Reachem, Moscow, Russia).

The glycine–nitrate precursors of LaCrO_3_ and LaMnO_3_ (LaCrGly, LaMnGly) were synthesized by the standard procedure. The reagents (nitrates of the metals and glycine) were dissolved in a minimal volume of water in a glass beaker and stirred for 15 min at room temperature. The quantities of the reagents, volumes of the water, and pH of the solutions are listed in Table 1. The aqueous solution of the reagents was heated on an IKA C-MAG HS4 digital plate (IKA, Staufen, Germany) for 2 to 3 h at 60 °C under constant stirring. The formed viscous gel was then dried in a vacuum box for 2 h at 60 °C. The resulting solid was ground into powder in a hand mortar and stored in an exicator over P_2_O_5_. Monometallic precursors (CrGly, MnGly) were also prepared by the same method to serve as model compounds (Table 1). The Gly/NO_3_ molar ratio was equal to 1 in both the monometallic and bimetallic precursors. A sample designated as GlyHNO_3_ was synthesized to serve as a reference compound for the analysis of the infrared spectra of the precursors. For its preparation, aqueous solutions of glycine and HNO_3_ were mixed in a mole ratio of 1:1. The rest of the procedure was analogous to the synthesis of the precursors.

The elemental stoichiometric coefficient (ϕ) was calculated by the standard procedure [27], taking into account the stoichiometry of Equations (1) and (2) as follows:

La(NO_3_)_3_ + Cr(NO_3_)_3_ + 6C_2_H_5_NO_2_ + 6O_2_ = LaCrO_3_ + 12CO_2_ + 15H_2_O + 6N_2_ (1)

La(NO_3_)_3_ + Mn(NO_3_)_2_ + 5C_2_H_5_NO_2_ + 11.5O_2_ = LaMnO_3_ + 10CO_2_ + 12.5H_2_O + 5N_2_ (2)

(3) $$\phi \;(\mathrm{LaCrGly})=\frac{n\left[2\cdot 4_{\left(C\right)}+5\cdot 1_{\left(H\right)}+0_{\left(N\right)}-2\cdot 2_{\left(O\right)}\right]}{w\left[3_{\left(La\right)}+3\left(0_{\left(N\right)}-3\cdot 2_{\left(O\right)}\right)]+y[3_{\left(Cr\right)}+3\left(0_{\left(N\right)}-3\cdot 2_{\left(O\right)}\right)\right]}=1.8$$

(4) $$\phi \;(\mathrm{LaMnGly})=\frac{n\left[2\cdot 4_{\left(C\right)}+5\cdot 1_{\left(H\right)}+0_{\left(N\right)}-2\cdot 2_{\left(O\right)}\right]}{w\left[3_{\left(La\right)}+3\left(0_{\left(N\right)}-3\cdot 2_{\left(O\right)}\right)]+y[2_{\left(Mn\right)}+2\left(0_{\left(N\right)}-3\cdot 2_{\left(O\right)}\right)\right]}=1.8$$

where *n*, *w*, and *y* are the number of moles of glycine, nitrate of lanthanum, and nitrate of chromium or manganese in Equations (1) and (2), respectively.

Thus, the combustion reactions of both LaCrGly and LaMnGly had the same value of the fuel/oxidizer ratio, ϕ. The combustion of the precursors was carried out in air using the following three combustion regimes: self-propagating high-temperature synthesis (SHS), volume combustion synthesis (VCS), and the traditional solution combustion synthesis (SCS).

### 2.2. Synthesis of Oxides in SHS

First, 150 mg of a dried powder of the precursor (LaCrGly, LaMnGly, CrGly, MnGly) were pressed into a pellet with a diameter of 5 mm and a thickness of ~1.5 mm at 70 bar using a PGR-10 laboratory desk-top hydraulic press (LabTools, Pokrovskaya, Russia). Then, the burning of the pellet was initiated within 1–3 s with the help of a butane–propane lighter, after which the source of the heat was removed and the formation of loose «serpentines» of the combustion products was observed, which were growing in length (Figure S1, Supplementary Materials). The combustion products of each individual pellet (23 and 29 pieces for LaMnO_3_ and LaCrO_3_, respectively) were mixed together to obtain an averaged product that was studied using a series of physicochemical methods.

The rate of combustion of the LaCrGly and LaMnGly pellets was determined as follows: the overall time of combustion of each pellet was measured with a stop watch (after a short process of initiation). The mass burning velocity expressed in mg/s was calculated for each pellet and the obtained values were averaged. In one series of experiments, the calculated values did not differ by more than 10 rel%.

### 2.3. Synthesis of Oxides in VCS

A dried powder of the precursor (LaCrGly, LaMnGly, CrGly, and MnGly) was poured into a quartz beaker to form a thin layer (with a thickness of about 1–1.5 mm) and the beaker was placed onto a pre-heated (to 500 °C) IKA C-MAG HS4 digital plate. During the heating, all powder ignited simultaneously to form a voluminous loose residue (Figure S2, Supplementary Materials). After the burning was complete, the heating was continued for another 2 to 3 min under constant stirring until no local sparking was observed.

### 2.4. Synthesis of Oxides in SCS

An aqueous solution of the reagents for the synthesis of LaCrGly and LaMnGly (Table 1) was evaporated for 1 to 2 h under constant stirring to the state of a viscous gel on an IKA C-MAG HS4 digital plate, which was pre-heated to 300 °C. Then, the temperature was raised to 500 °C and within several minutes an explosion-like ignition of the sample took place as in the VSC regime. After the combustion was complete, the heating continued for another 2 to 3 min under constant stirring until no local sparking was observed.

### 2.5. Methods of Investigation

Attenuated total reflection infrared spectroscopy (ATR FTIR) was performed on an Agilent Cary 600 (Agilent Technologies, Santa Clara, CA, USA) spectrometer equipped with a Gladi ATR attachment (PIKE Technologies, Madison, WI, USA) in the range from 300 to 4000 cm^−1^ without a pretreatment of the samples.

The thermal analysis of the precursors and oxides was performed on a Netzsch STA 449 C Jupiter instrument (NETZSCH, Selb, Germany) equipped with a DTA/TG holder in the temperature range of 20–400 °C under a flow of helium or purified and dry air. The heating rate of the samples was 10 °C/min, and the weight of the samples was 10 or 20 mg.

High-resolution transmission electron microscopy (HRTEM) images were obtained on a JEM-2010 microscope (Jeol, Akishima, Japan) (with an accelerating voltage of 200 kV and a resolution of 0.14 nm). The images were analyzed and filtered using the Digital Micrograph program (GATAN, Pleasanton, CA, USA). A suspension of perovskite particles in ethanol was deposited onto a copper support with an ultrasonic disperser.

The X-ray diffraction (XRD) analysis of the products of combustion was performed on a D8 Advance diffractometer (Bruker AXS GmbH, Karlsruhe, Germany) equipped with a linear detector Lynexeye. The XRD patterns were obtained in the 2θ range from 15° to 80° with a step of 0.05°, and the time of accumulation was 3 s in each point. CuK_α_ radiation (λ = 1.5418 Å) was used. The phases were identified using the following data: LaCrO_3_ (PDF 24–1016), LaMnO_3_ [PDF 50–297], La_2_O_2_CO_3_ (PDF 37–804, PDF 48–1113), La_2_O(CO_3_)_2_·(PDF 41–672), Cr_2_O_3_ (PDF 38–1479), CrO_2_ (PDF 9–332), Mn_3_O_4_ (PDF 20–0734), and MnO (PDF 07–0230). The average coherent scattering regions (CSR^1^) were determined by the widely used Scherer formula from the peaks: 101 for LaMnO_3_, 110 for LaCrO_3_, 112 for Mn_3_O_4_, 022 for MnO, and 012 for Cr_2_O_3_. The relative error in determining the CSR^1^ is about ±10%. Additionally, Rietveld refinement for quantitative analysis was carried out using the Topas V.4.2 software package. The instrumental broadening was described with metallic silicon as a reference material. Size-strain analysis was performed using the double-Voigt approach. In this case, CSR^2^ was calculated using LVol-IB values (i.e., volume averaged column height calculated from the integral breadth) [41]. The CSR^2^ and strain rates and their determination accuracy are shown in Table S1 (Supplementary Materials).

The specific surface area (S_BET_) was determined by desorption of argon using a Sorbi-M instrument (Meta, Novosibirsk, Russia). The relative error in determining the specific surface area is about ±6%.

The contents of La, Mn, and Cr in the precursors were determined by inductively coupled plasma atomic emission spectrometry on an Optima 4300 DV instrument (PerkinElmer, Waltham, MA, USA). The contents of C, H, and N were determined on an automatic CHNS analyzer EURO EA 3000 (Euro Vector S.p.A., Castellanza, Italy). The samples (0.5–2 mg) were combusted in a vertical reactor in the dynamic regime at 1050 °C in a flow of He with added O_2_. The absolute error of concentration measurement is in the range of ±0.3–0.5 wt%. From the obtained data, the compositions of precursors were calculated and compared with the theoretical ones (Table 1).

## 3. Results and Discussion

### 3.1. ATR FTIR Study of Dry Precursors

The synthesis of MnGly, CrGly, LaMnGly, and LaCrGly precursors was performed from an acidic medium (Table 1). During their preparation, a certain amount of nitric acid may form as a result of hydrolysis of the metal nitrate solution and as a result of glycine interaction with the cations of the metals to form complex compounds. It is known that glycine also interacts with nitric [42] and other acids [43]. A comparison of the ATR FTIR spectrum of GlyHNO_3_ (Table S2, Supplementary Materials) with the spectra of the compounds of glycine with HClO_4_ and HBF_4_ [43] revealed their close similarity. A distinctive spectral feature of these compounds is the presence of strong absorption bands (a.b.) characteristic of the vibrations of the functional groups υ(C=O) and υ(C–OH) (Table S2). Thus, the spectrum of GlyHNO_3_ is substantially different from the spectrum of the initial glycine (Table S2), which has the structure of a zwitterion [42].

**Figure 1:**
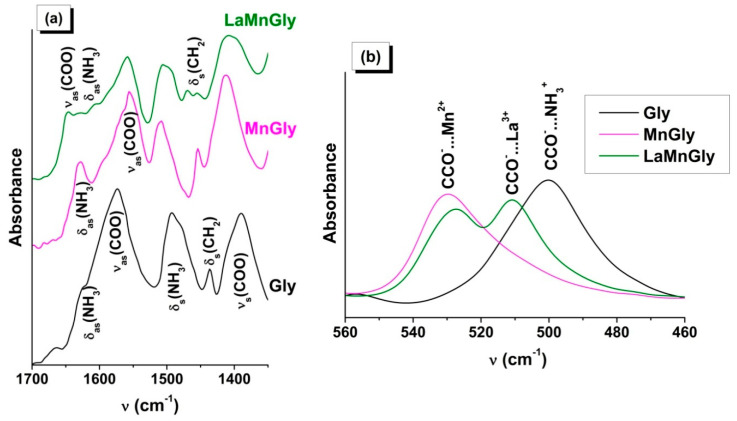
(**a**) ATR FTIR spectra of Gly, MnGly, and LaMnGly. (**b**) The region of deformational vibrations of the CCO groups.

In the ATR FTIR spectra of the dried MnGly and LaMnGly precursors, no a.b. belonging to GlyHNO_3_ or the initial glycine were present (Figure 1a, Table S2). From the comparison of these data, it follows that, in the case of both the monometallic MnGly and bimetallic LaMnGly, there was a change in the position of the a.b. belonging to the valence vibrations (ν_as_(COO), ν_s_(COO)) and the deformational vibrations (δ(COO), ω(COO), δ(CCO)) of the glycine carboxyl groups. There were broad absorption bands of a low intensity in the region of 360–280 cm^−1^ characteristic of the metal–oxygen bond (Table S2).

From this, it follows that in the acidic medium the interaction of glycine with the cations of manganese and lanthanum takes place via the carboxyl group [44,45]. It can be suggested that the bimetallic precursor consists of a mixture of compounds. This is indicated by the presence in the spectrum of the bimetallic precursor of a.b. belonging to MnGly and by the appearance of new absorption bands (Figure 1a,b and Table S2) attributable to the valence and deformational vibrations of the COO groups interacting with the nitrate of lanthanum. The appearance of ν_as_(COO) at a higher frequency and the increase in the value of ∆ = ν_as_(COO) − ν_s_(COO) to 237–240 cm^−1^ are consistent with the IR and XRD data [44] for the compound La(Gly)_3_(ClO_4_)_3_·2H_2_O (Gly = NH_3_^+^CH_2_COO^−^) forming in an acidified aqueous solution as a result of interaction of lanthanum salt with glycine.

**Figure 2:**
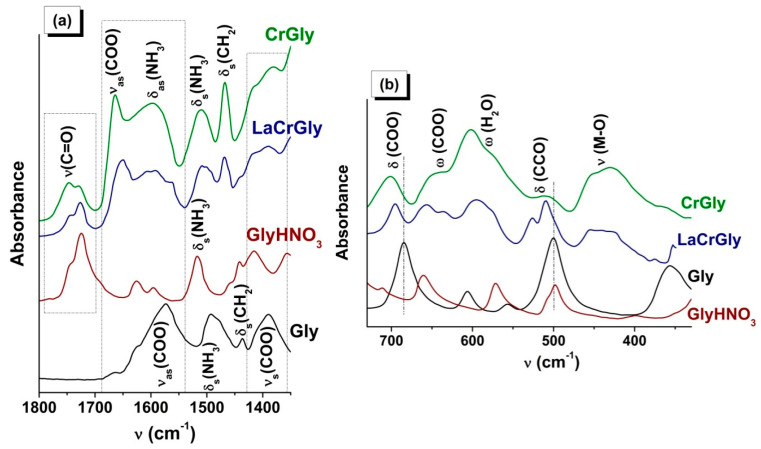
(**a**) ATR FTIR spectra of Gly, GlyHNO_3_, CrGly, and LaCrGly. (**b**) The region of deformational vibrations of the COO and CCO groups.

Figure 2 shows ATR FTIR spectra of the products of interaction of glycine with the nitrate of chromium and with the nitrate of chromium together with the nitrate of lanthanum. As in the previous case, there were changes in the state of the glycine carboxyl groups indicating their interaction with ions of chromium and lanthanum (Table S2). However, in the case of both the monometallic CrGly and bimetallic LaCrGly, the type of interaction was different from that in the above-described compounds of manganese. In the spectrum of CrGly, the shift of the a.b. of the valence vibrations of the COO groups was considerably greater, and several additional a.b. appeared in the region of the deformational vibrations of the COO groups. Most of the a.b. were broadened. In addition to this, the sample was found to contain some amounts of water and a compound similar to GlyHNO_3_ (Figure 2, Table S2). The formation of the latter appears to be associated with the lower values of pH in the initial solution of reagents (Table 1). Comparison of the spectrum of the prepared LaGly precursor with the literature data indicated its similarity with the spectrum of the complex compound [Cr_3_O(Gly)_6_(H_2_O)_3_](NO_3_)_7_·3H_2_O (I) synthesized in an acidic medium, where Gly = NH_3_^+^CH_2_COO¯ [46,47]. Characteristic of this complex is the presence in its spectrum of a.b. at 1663, 636, and 450 cm^−1^ attributed by the authors [46] to the ν_as_(COO), δ(COO), and υ(Cr–O) vibrations, respectively.

This allows us to suggest that, in the synthesis of CrGly, glycine preferably interacts with chromium nitrate to form a compound with a composition and structure close to (I). As in the case of (I), the CrGly precursor has a green color, which confirms the Cr–O bonds’ formation taking place as a result of interaction of the glycine carboxyl group with chromium cations.

The addition of the nitrate of lanthanum to CrGly led to a considerable reduction in the relative intensity of all a.b. and their broadening (Figure 2). Moreover, there was splitting of the a.b. of the deformational and valence vibrations of the NH_3_^+^ and CH_2_ groups, probably as a result of an intermolecular interaction. The analysis of the shapes and positions of the a.b. (Table S2) shows that, with the addition of lanthanum nitrate to MnGly and CrGly, in both cases there appeared a high-frequency band (1646, 1648 cm^−1^) in the region of ν_as_(COO) vibrations. These changes may indicate a similar type of interaction of La(NO_3_)_3_ with glycine in LaMnGly and LaCrGly precursors.

### 3.2. Thermal Analysis of Dry Precursors

**Figure 3:**
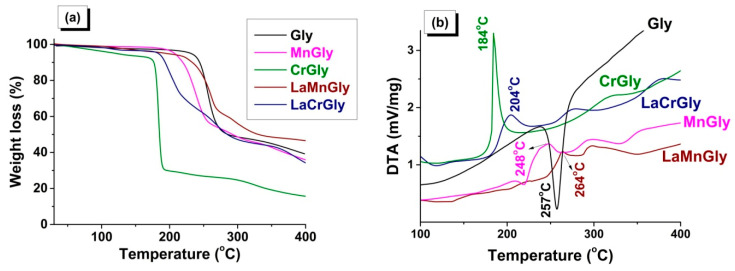
Thermal analysis of Gly, MnGly, CrGly, LaMnGly, and LaCrGly: (**a**) TG curves; (**b**) DTA curves (He, 20 mg, 10 °C/min).

In Figure 3, the results of the thermal analysis obtained in an inert atmosphere for MnGly, CrGly, LaMnGly, and LaCrGly are compared with the thermolysis of initial glycine. It was shown that the thermolysis of glycine is a one-step endothermic process with the maximum at 257 °C (Figure 3b). On the contrary, the thermolysis of all precursors is shown as exothermic redox multi-step processes, which appears to be associated with the formation of intermediate products as was discussed in [10].

It was shown that, among the studied precursors, CrGly turned out to be the most reactive compound characterized by the lowest temperature of the onset of the decomposition (~170 °C), a fast rate of the process, and a high extent of gasification at 184 °C (Figure 3b). We believe this was due to the high reactivity of (I), which, according to ATR FTIR, is the main structural element of this precursor. The onset of the MnGly decomposition was shifted by ~50 °C towards higher temperatures, and the rate of its thermolysis and the extent of gasification were smaller than in the case of the CrGly precursor (Figure 3). The addition of lanthanum to CrGly and MnGly decreases the rate of its thermolysis and the extent of gasification. It can be seen that the temperatures of their maxima on the DTA curves shift from 184 to 204 °C in the case of LaCrGly and from 248 to 264 °C in the case LaMnGly (Figure 3b). However, the LaCrGly decomposition takes place at lower temperatures as compared with LaMnGly.

### 3.3. Effect of Combustion Regime of Precursors on the Characteristics of the Forming Products

**Figure 4:**
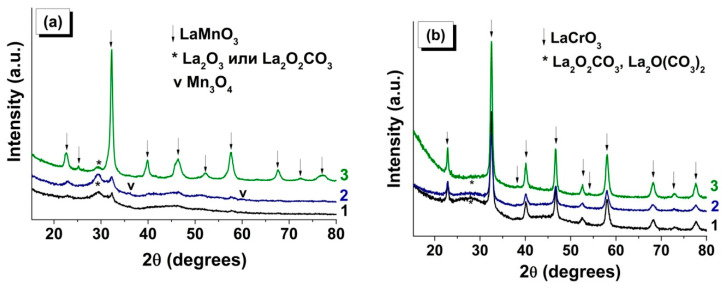
XRD of combustion products of (**a**) LaMn- and (**b**) LaCr-precursor for the following regimes: (1) solution combustion synthesis (SCS) (from solution), (2) volume combustion synthesis (VCS) (from powder), and (3) self-propagating high-temperature synthesis (SHS) (from pellet).

Figure 4 shows X-ray patterns of the products of combustion of LaMnGly and LaCrGly produced in the different regimes: (1) SCS, the traditional procedure using the solution of initial reagents, (2) VCS using the dried powder of the precursor, and (3) SHS using the pressed pellet of the precursor. In Table 2 are listed the values of the coherent scattering region (CSR) of the oxide phases and the specific surface area of the products forming as a result of combustion.

It is seen that the regime of combustion and the nature of the transition metal (Cr, Mn) have a substantial influence on the characteristics of the forming oxide product. Thus, the diffraction patterns of the products of LaMnGly combustion (Figure 4a) formed by traditional SCS and by VCS show, apart from the broadened peaks belonging to a poorly crystallized phase of the perovskite, noticeable broad peaks in the 25–32° 2θ-region. Note that it is the region of the most intense peaks of X-ray patterns of La_2_O_2_CO_3_ and La_2_O(CO_3_)_2_, whose crystal structure is close to that of La_2_O_3_. A well-crystallized phase of LaMnO_3_ (Table 2) was only formed under the SHS conditions (Figure 4a). In this case, the diffraction pattern showed a considerable decrease in the intensity in the 25–32° 2θ-region attributed to lanthanum carbonates. The use of different approaches to CSR estimation gave similar results (Table 2). For SHS samples, the values of CSR^1^ and CSR^2^ were 11 and 10 nm, respectively.

A different situation was observed for the combustion products of LaCrGly precursors (Figure 4b). A crystalline phase of LaCrO_3_ was formed in all combustion regimes, but there was a general trend that, with the change from SCS and VCS to SHS, there was an increase in the value of CSR^1^ and CSR^2^ of the perovskite phase while the content of lanthanum carbonate phases decreased (Table 2). Note that, in the case of the traditional solution combustion synthesis (SCS), the halo in the region of the main peaks of lanthanum carbonates was more pronounced (Figure 4b).

Analysis of XRD data for the combustion products of monometallic precursors (Table 2) also reveals the role of combustion regimes in determining the phase composition and the dispersion of the forming oxide phases (Cr_2_O_3_, Mn_3_O_4_, and MnO). This is a further confirmation of the general observation that the use of SHS increases CSR^1^ and CSR^2^ of the oxides. We believe that this was associated with the fact that in SHS the heat is concentrated in a narrow reaction zone between the forming product and the precursor that did not enter into the reaction. As shown above, this also leads to a decrease in the content of the thermally stable phases of lanthanum carbonates (in the case of combustion of LaMnGly and LaCrGly precursors) [48] and an increased fraction of the high-temperature MnO phase in the products of combustion of the MnGly precursor.

**Figure 5:**
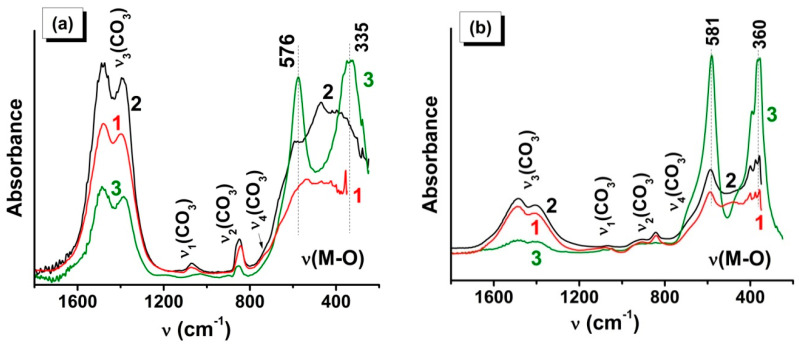
ATR FTIR spectra of combustion products of (**a**) LaMn and (**b**) LaCr precursor for the following regimes: (1) SCS (from solution), (2) VCS (from powder), and (3) SHS (from pellet).

The ATR FTIR data (Figure 5) in the region of the vibrations of the carbonate groups and metal–oxygen bonds are in full agreement with XRD. Note that the absorption bands at 576 and 335 cm^−1^ (Figure 5a) and at 580 and 360 cm^−1^ (Figure 5b) confirm the formation of LaMnO_3_ and LaCrO_3_, respectively [49,50]. The presence in the spectra of the SCS- and VCS-derived samples of LaMnO_3_ of a broad poorly structured spectral line in the region of valence metal–oxygen vibrations indicates a low extent of crystallinity of the perovskite phase and shows that a heat treatment is necessary to complete the process of the phase formation (Figure 5b).

The observed positions and intensities of the absorption bands due to the carbonate anion vibration (Figure 5) are in good agreement with the spectra for La_2_O_2_CO_3_ [51,52]. From the analysis of the absorption intensities in the regions of metal–oxygen and carbonate anion vibrations, it can be concluded that the formation of carbonate impurities was more typical for the combustion of LaMnGly (Figure 5a), especially in the SCS and VCS regimes, whereas the sample LaCrO_3_ synthesized in the SHS regime (Figure 5b), along with the high values of CSR (Table 2), shows the lowest content of carbonate impurities.

**Figure 6:**
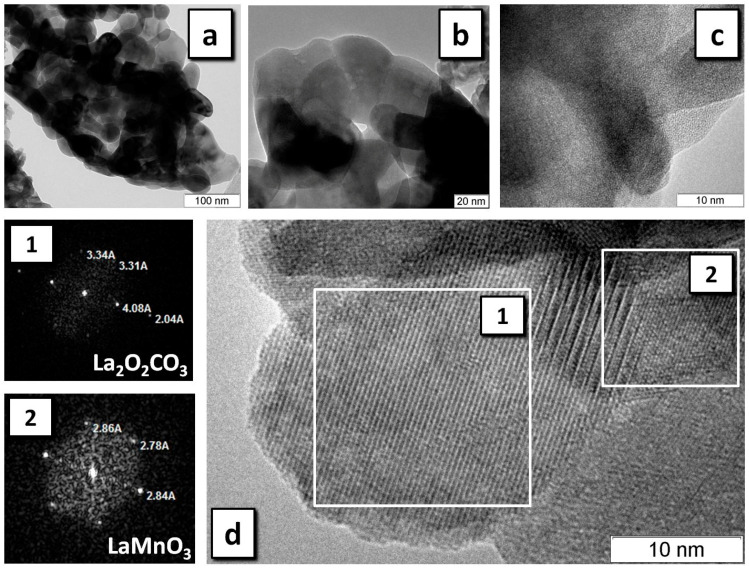
HRTEM of LaMnO_3_ obtained in the SHS regime: LaMnO_3_ phase with resolution of (**a**) 100 nm, (**b**) 20 nm; (**c**) amorphous phase; (**d**) the selected areas and their diffraction patterns (1), (2).

**Figure 7:**
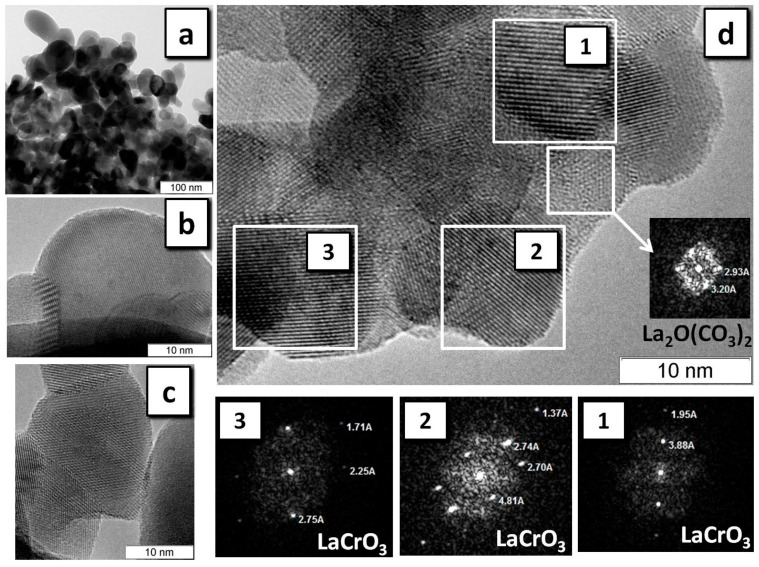
HRTEM of LaCrO_3_ obtained in the SHS regime: LaCrO_3_ phase with resolution of (**a**) 100 nm and (**b**,**c**) 10 nm for different particles; (**d**) the selected areas and their diffraction patterns (1), (2), and (3).

Figure 6 and Figure 7 show the HRTEM data for LaMnO_3_ and LaCrO_3_ prepared by SHS, the most productive regime for the synthesis of the perovskite phase. Thus, in LaMnO_3_, selected areas of the particles were found consisting of the LaMnO_3_ crystalline phase (Figure 6a,b) and representing an amorphous phase of a non-carbon nature (Figure 6c). Analysis of the diffraction patterns of some selected areas showed that, along with the phase of the perovskite, the sample often shows the presence of La_2_O_2_CO_3_ (Figure 6d) and, less frequently, of La_2_O(CO_3_)_2_. Only a single area was found with a structure attributable to Mn_5_O_8_. On the whole, the size of the particles in the observed agglomerates coincides with CSR for LaMnO_3_ determined by XRD (Table 2).

The phase purity of the LaCrO_3_ sample is substantially different from that of LaMnO_3_ according to HRTEM data (Figure 7). Analysis of the diffraction patterns of selected areas of individual particles shows that the sample mainly consists of well-crystallized LaCrO_3_ (Figure 7d), and no zones of amorphous phases were found. The frequency of occurrence of areas with the structure of La_2_O_2_CO_3_ or La_2_O(CO_3_)_2_ is substantially smaller than in the case of LaMnO_3_.

**Figure 8:**
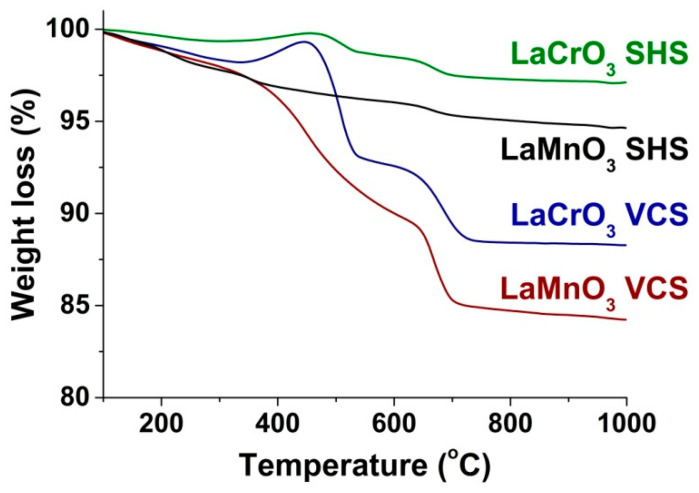
Thermogravimetric analysis of LaMnO_3_ and LaCrO_3_ obtained in the different combustion regimes (air, 20 mg, 10 °C/min).

Figure 8 shows the thermal analysis data for the perovskites prepared by VCS and SHS. It can be seen that the SHS samples show rather small losses of mass of below 5% during their heating in a flow of air, which is consistent with their low contents of impurity phases as determined by ATR FTIR and HRTEM. We believe that, in this case, the loss of the mass was associated with the decomposition of lanthanum carbonates. Similar but more intense stages of decomposition are observed in the TG curves of the VCS samples, which confirms that they were caused by decomposition of the main impurity, i.e., the carbonate phases of lanthanum. Both in the VCS and SHS regimes, the loss of mass in the case of LaMnO_3_ was greater than in the case of LaCrO_3_, which is in agreement the XRD, ATR FTIR, and HRTEM results about the content of the carbonate phase impurity. The insignificant increase in the mass of the LaCrO_3_ samples around 450 °C appears to indicate their interaction with oxygen or CO_2_.

## 4. Conclusions

Table 3 presents the literature data on the glycine–nitrate combustion synthesis of LaMnO_3_ and LaCrO_3_. The elemental stoichiometric coefficient (ϕ) of the precursor, its combustion conditions, and additional calcination are the main factors determining the formation of the crystalline phase of perovskite. It is worth noting that these data (Table 3) are difficult to analyze since all the characteristics of the precursor and details of the combustion stage should be considered. However, they are not always indicated. For example, LaMnO_3_ was synthesized at ϕ ~ 1 under solution combustion synthesis (SCS) at external heating of both 350 [34] and 700 °C [27,53] without additional calcination. However, the specific surface area of the combustion product obtained at 350 °C was smaller (16.6 m^2^/g) than in case of the product obtained at 700 °C (24 m^2^/g [53]). Crystalline LaMnO_3_ was also formed upon additional calcination of the amorphous combustion product at 700 and 900 °C [27,30,34,54]. Note that the water evaporation from the precursors and the use of dry gel (ϕ ≤ 1) did not allow the synthesis of the crystalline LaMnO_3_ at reduced heating temperature (300–400 °C) in one step without additional calcination [30,54]. It was possible in the case of combustion of the dry gel precursor with ϕ = 1.8 [54]. At the same temperature (350 °C), SCS of such a fuel-rich precursor did not lead to the formation of the well-crystalline LaMnO_3_ phase [34]. Only an increase in temperature to 700 °C gave this result [27]. As for the synthesis of crystalline LaCrO_3_ (Table 3), we found information about successful calcination-free syntheses (ϕ = 1, 2) in the case of SCS at 250 °C [31] and at a linear temperature rise to 800 °C [55]. In contrast, the combustion of dry gel (ϕ = 1) at 300 °C led to the formation of the mixture of monometallic oxygen-containing crystalline phases [30]. Table 3 shows that the high values of specific surface area for LaMnO_3_ (37 m^2^/g [27]) and LaCrO_3_ (41 m^2^/g [31]) were obtained in fuel-rich conditions at ϕ = 1.5 and ϕ = 2, respectively. It should be noted that insufficient attention is given to the formation of the thermally stable lanthanum carbonate impurities. Our previous studies show that it is the main problem of the calcination-free synthesis of La-containing complex oxides using combustion methods [39,40].

From the results of this study, one may conclude that the self-propagating high-temperature synthesis (SHS) using a pellet of a dried glycine–nitrate precursor carried out under fuel-rich conditions (ϕ = 1.8) is the most effective calcination-free regime allowing the preparation of perovskites (LaMnO_3_ and LaCrO_3_). The external heat is necessary for a short stage of the initiation of the combustion process. Then, the combustion of the pellet continues at room temperature. The study of the combustion products using a series of methods (XRD, ATR FTIR, HRTEM, thermal analysis) has suggested that with the SHS regime higher temperatures were developed in the reaction zone, which led to increased yields of the crystalline product with smaller contents of the thermally stable lanthanum carbonate phases and a high specific surface area (Table 3, 32 and 27 m^2^/g for LaMnO_3_ and LaCrO_3_, respectively). No substantial differences were found between the traditional SCS and volume combustion synthesis (VCS), which appears to indicate that in SCS the ignition of the gel takes place under the conditions when most of the water was evaporated from the precursor.

However, the analysis of the obtained results showed that the nature of the transition metal (Mn, Cr) has a significant influence on the yield of the perovskite phase and on its dispersion and purity at the same fuel/oxidant ratio. For comparison, estimated values of the adiabatic temperature of combustion for LaCrGly (1) and LaMnGly (2) (see Supplementary Materials, Equations (1S), Tables S3 and S4), measured values of the rates of their combustion, the temperatures of the onset of their decomposition (based on the thermal analysis data), and the composition of the forming products are given in Table 4. It is clear that the estimated value of the adiabatic temperature is considerably higher than the actual temperature as some of the heat is lost in the interchange with the environment. Nevertheless, this value allows a comparison to be made between the two systems. Such an approach has generally been employed in discussions on burning processes [35].

Analysis of the results in Table 4 allows an explanation of the reasons for the higher yield of the crystalline phase and the small content of carbonate impurities in the product formed in SHS with the LaCrGly, namely the low temperature of the onset of the thermolysis, the high value of the adiabatic temperature, and the high rate of combustion. We believe that these characteristics are interrelated and are determined by the thermodynamics and kinetics of the combustion process.

## Figures and Tables

**Table 1:** Data on the preparation of glycine–nitrate precursors.

Samples	Reagents	Quantity	pH	Theoretical Composition ^1^	Determined Composition ^2^
CrGly	Cr(NO_3_)_3_∙9H_2_O	2.0 g	3.1	CrC_6_H_15_N_6_O_15_	CrC_6_H_16.5_N_5.8_O_15.8_
	Gly	1.125 g			
	H_2_O	10 mL			
MnGly	Mn(NO_3_)_2_∙4H_2_O	1.255 g	4.5	MnC_4_H_10_N_4_O_10_	MnC_4_H_9.7_N_3.9_O_9.9_
	Gly	0.75 g			
	H_2_O	10 mL			
LaCrGly	La(NO_3_)_3_∙6H_2_O	2.165 g	2.9	LaCrC_12_H_30_N_12_O_30_	LaCr_1.3_C_12_H_32.7_N_11.9_O_33.8_
	Cr(NO_3_)_3_∙9H_2_O	1.255 g			
	Gly	1.875 g			
	H_2_O	10 mL			
MnCrGly	Mn(NO_3_)_2_∙4H_2_O	2.165 g	4.1	LaMnC_10_H_25_N_10_O_25_	LaMn_1.1_C_10_H_26.4_N_9.8_O_27.6_
	Cr(NO_3_)_3_∙9H_2_O	2.0 g			
	Gly	2.25 g			
	H_2_O	10 mL			

^1^ The theoretical composition of the precursor under assumption that water was completely removed by the drying. ^2^ The values were calculated based on the data of the elemental analysis; the mass fraction of oxygen was estimated as the difference between 100% and mass fractions of metals, carbon, hydrogen, and nitrogen.

**Table 2:** Phase composition and specific surface area for combustion products formed in the different regimes. The standard deviation is indicated in brackets.

Sample	Phase Composition	S_BET_ (m^2^/g)	CSR^1^ (nm)	CSR^2^ (nm)
Monometallic precursors				
MnGly SHS	Mn_3_O_4_ (75 wt%)	−	76(7)	75(9)
	MnO (25 wt%)		72(7)	75(9)
MnGly VCS	Mn_3_O_4_ (99 wt%)	−	31(3)	25(3)
	MnO (1 wt%)		43(4)	−
CrGly SHS	Cr_2_O_3_	−	41(4)	43(5)
	traces of CrO_2_		−	−
CrGly VCS	Cr_2_O_3_	−	26(3)	33(6)
	traces of CrO_2_		−	−
Bimetallic precursors				
LaMnGly SHS	LaMnO_3_	32(2)	11(1)	10(1)
	traces of La_2_O_2_CO_3_ or La_2_O(CO_3_)_2_		−	−
LaMnGly VCS	LaMnO_3_	37(2)	4(4)	−
	traces of La_2_O_2_CO_3_ or La_2_O(CO_3_)_2_		−	−
	Mn_3_O_4_		−	−
LaCrGly SHS	LaCrO_3_	27(2)	35(4)	43(5)
LaCrGly VCS	LaCrO_3_	38(2)	29(3)	30(4)
	traces of La_2_O_2_CO_3_ or La_2_O(CO_3_)_2_		−	−

**Table 3:** Synthesis of LaMnO_3_ and LaCrO_3_ perovskites using the glycine–nitrate combustion method.

Phase	Precursors	Combustion Mode	ϕ	Calcination	XRD Phase Composition	S_BET_ (m^2^/g)	CSR (nm)	Ref.
LaMnO_3_	La(NO_3_)_3_ Mn(NO_3_)_2_ Glycine	Solution Oven 350 °C	0.7	−	Amorphous, residual carbon	15.2	−	[34]
				900 °C, 1 h	LaMnO_3_	8–10	−	
			1.1	−	LaMnO_3_	16.6	−	
				900 °C, 1 h	LaMnO_3_	8–10	−	
			1.7	−	LaMnO_3_, traces LaONO_3_, residual carbon	24.8	−	
				900 °C, 1 h	LaMnO_3_	8–10	−	
LaMnO_3_	La(NO_3_)_3_ Mn(NO_3_)_2_ Glycine	Pellet Local ignition RT	1.8	−	LaMnO_3_, traces of La_2_O_2_CO_3_, La_2_O(CO_3_)_2_	32	11	In this work
LaMnO_3_	La(NO_3_)_3_ Mn(NO_3_)_2_ Glycine	Solution Furnace 700 °C	0.6	−	Amorphous, La_2_O_2_CO_3_, La_2_O(CO_3_)_2_	22	−	[27]
				700 °C, 24 h	LaMnO_3_	16	−	
			1	−	LaMnO_3+y_	18	28	
			1.5	−	LaMnO_3+y_	37	20	
LaMnO_3_	La(NO_3_)_3_ Mn(NO_3_)_2_ Glycine	Gel Hot plate 10 °C/min	1	−	LaMnO_3_	22	33	[56]
				400 °C	LaMnO_3_	20	38	
				600 °C	LaMnO_3_	23	47	
				800 °C	LaMnO_3_	9.1	33	
				1000 °C	LaMnO_3_	2.6	130	
LaMnO_3_	La(NO_3_)_3_ Mn(NO_3_)_2_ Glycine	Solution Oven 700 °C	1	−	LaMnO_3.16_	24	−	[53]
				700 °C, 24 h flowing air (N_2_/O_2_=4/1)	LaMnO_3.16_	16	−	
LaMnO_3_	La(NO_3_)_3_ Mn(NO_3_)_2_ Glycine	Gel Heater 300–400 °C	0.6	−	Amorphous	−	−	[54]
				10 °C/min, 700 °C, 4 h	LaMnO_3_	−	500 - 1000	
			1	−	LaMnO_3_ weak crystallinity	−	−	
				10 °C/min, 700 °C, 4 h	LaMnO_3_	−	−	
			1.8	−	Amorphous	−	−	
				10 °C/min, 700 °C, 4 h	LaMnO_3_	−	< 100	
LaMnO_3_	La(NO_3_)_3_ Mn(NO_3_)_2_ Glycine	Gel Muffle furnace 300 °C	1	−	Amorphous	−	−	[30]
				10 °C/min, 900 °C, 6 h, flowing air	LaMnO_3_	−	22.4	
LaCrO_3_	La(NO_3_)_3_ Cr(NO_3_)_2_ Glycine		1	−	La_2_O(CO_3_)_2_, Cr_5_O_12_, Cr_2_O_3_, Cr_3_O_4_	−	−	
				10 °C/min, 900 °C, 6 h, flowing air	LaCrO_3_, traces of La_2_O(CO_3_)_2_	−	22.3	
LaCrO_3_	La(NO_3_)_3_ Cr(NO_3_)_2_ Glycine	Solution Hot plate 250 °C	0.5	−	Amorphous, unreacted nitrates	35	−	[31]
			1	−	LaCrO_3_	16	26	
			2	−	LaCrO_3_, LaCrO_4_	33	14	
	La(NO_3_)_3_ (NH_4_)_2_Cr_2_O_7_ Glycine		0	−	La_2_CrO_6_, La_2_O_3_, Cr_2_O_3_	−	−	
			0.5	−	LaCrO_4_,unreacted nitrates	5.6	59	
			1	−	LaCrO_3_ *Pnma*	29	15	
			2	−	LaCrO_3_	41	15	
LaCrO_3_	La(CH_3_COO)_3_ Cr(NO_3_)_2_ Glycine	Solution 10 °C/min 800 °C, 0.5 h	0	−	La_2_CrO_6_, LaCrO_3_	6	150	[55]
			1	−	LaCrO_3_	7.3	119	
			2	−	LaCrO_3_	6.5	135	
			3	−	LaCrO_3_, La_2_CrO_6_	9.5	94	
			4	−	LaCrO_3_, La_2_CrO_6_	6.6	135	
			5		LaCrO_3_, La_2_CrO_6_	7.1	124	
LaCrO_3_	La(NO_3_)_3_ Cr(NO_3_)_2_ Glycine	Pellet Local ignition RT	1.8	−	LaCrO_3_	27	34	In this work

**Table 4:** Comparison of SHS combustion of LaMnGly and LaCrGly precursors.

Parameter	LaMnGly	LaCrGly
T_ad._ (°C)	1562	2402
Pellet combustion rate (mg/s)	2.4	10.1
Start of thermolysis (°C)	245	180
*CSR* of LaMO_3_, M = Mn, Cr (nm)	11	35
Additional characteristics according to XRD, ATR FTIR, HRTEM	Coexistence of amorphous phase, high carbonate content	Crystalline product, low carbonate content

## Supplementary Materials

The following are available online at https://www.mdpi.com/1996-1944/13/22/5091/s1, Figure S1: The synthesis of LaMnO_3_ in the SHS regime: (**a**) ignition of precursor pellet; (**b**) and (**c**) layer-by-layer self-propagating high-temperature synthesis; (**d**) combustion product. Figure S2: The synthesis of LaMnO_3_ in the VCS regime: (**a**) thin layer of precursor powder at the glass bottom; (**b**) volume combustion synthesis at 500 °C heating; (**c**) and (**d**) combustion product. Table S1: Phase composition, coherent scattering domain size, and strain rate for combustion products formed in the different regimes calculated using the Topas V.4.2 software package. The standard deviation is indicated in brackets. Table S2: The comparison of ATR FTIR spectra of the Mn and Cr-containing glycine–nitrate precursors. Table S3: Enthalpy and heat capacity data used to estimate the adiabatic temperature. Table S4: Estimated thermodynamic data for combustion of precursors.
