# Supplementary material for: The Formation of Perovskite during the Combustion of an Energy-Rich Glycine–Nitrate Precursor
Source: Materials (Basel). 2020 Nov 11;13(22):5091. doi: 10.3390/ma13225091 (PMC7696830; doi:10.3390/ma13225091)
Supplement: Supplementary file 1 [file materials-13-05091-s001.pdf]

Supplementary Materials

The formation of perovskite during combustion of an energy–rich glycine–nitrate precursor

Oksana V. Komova, Svetlana A. Mukha, Anna M. Ozerova, Galina V. Odegova, Valentina I. Simagina, Olga A. Bulavchenko, Arcady V. Ishchenko and Olga V. Netskina *

* Correspondence: netsina@catalysis.ru; Tel.: +7–383– 330–74–58 (O.V.N.)

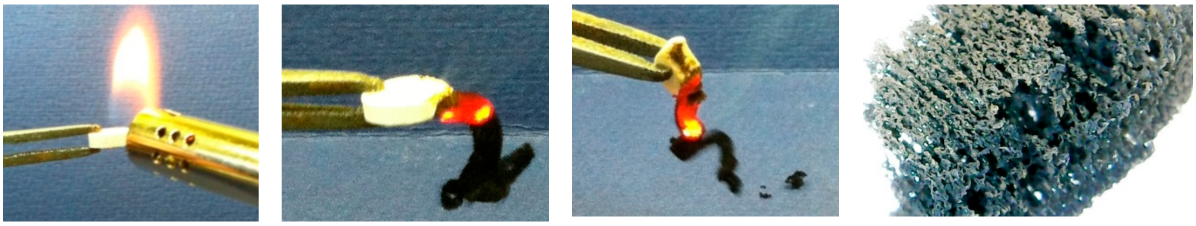

(**a**) (**b**) (**c**) (**d**)

Figure S1. The synthesis of $LaMnO_3$ in the SHS regime: (**a**) ignition of precursors pellet; (**b**) and (**c**) layer by layer self–propagating high temperature synthesis; (**d**) combustion product.

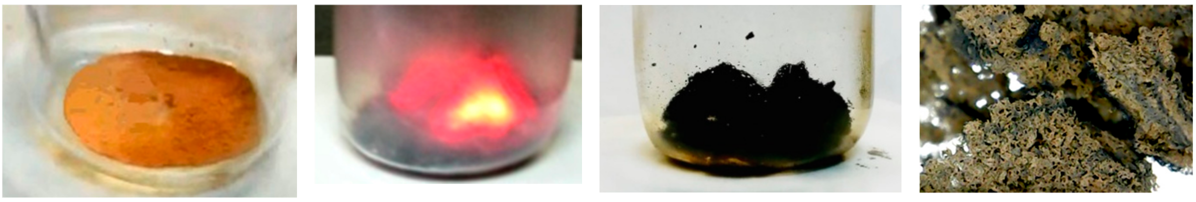

(**a**) (**b**) (**c**) (**d**)

Figure S2. The synthesis of $LaMnO_3$ in the VCS regime: (**a**) thin layer of precursors powder at the glass bottom; (**b**) volume combustion synthesis at 500 °C–heating; (**c**) and (**d**) combustion product.

Table S1. Phase composition, coherent scattering domain size, and strains rate for combustion products formed in the different regimes calculated using the software package Topas V.4.2. The standard deviation is indicated in brackets.

Sample	Phase composition	Strains (%)	CSR[2] (nm)
MnGly SHS	Mn_3O_4 (75 wt%)	0.07(0.01)	75(9)
	MnO (25 wt%)	0.04(0.01)	75(9)
MnGly VCS	Mn_3O_4 (99 wt%)	0.26(0.02)	25(3)
	MnO (1 wt%)	–	–
CrGly SHS	Cr_2O_3	0.12(0.01)	43(5)
	traces of CrO_2	–	–
CrGly VCS	Cr_2O_3	0.18(0.01)	33(6)
	traces of CrO_2	–	–
LaMnGly SHS	$LaMnO_3$	0.24(0.02)	10(1)
	traces of $La_2O_2CO_3$ or $La_2O(CO_3)_2$	–	–
LaMnGly VCS	$LaMnO_3$<br>traces of $La_2O_2CO_3$ or $La_2O(CO_3)_2$<br>Mn_3O_4	–	–
LaCrGly SHS	$LaCrO_3$	0.26(0.02)	43(5)
LaCrGly VCS	$LaCrO_3$	0.13(0.03)	30(4)
	traces of $La_2O_2CO_3$ or $La_2O(CO_3)_2$	–	–

Table S2. The comparison of ATR FTIR spectra of the Mn, Cr–containing glycine–nitrate precursors.

Vibration	Gly	$GlyHNO_3$	CrGly	LaCrGly	MnGly	LaMnGly
ν(C=O)	–	1740sh	1749w	1744w	–	–
	–	1722s	1719w	1726w	–	–
	–	–	–	–	–	–
$\delta_{as}(NH_3)$	1622sh	1624m	1593m	1589m	1627m	1627m
				1605m		1603m
				1582m		
				1571m		
$\nu_{as}(COO) + \delta(H_2O)$	–	–	1663m	1661	–	–
	–		–	1648	–	1646
	–		–	–	–	–
$\nu_{as}(COO)$	1573s			–	1555m	1557m
$\delta_s(NH_3)$	1492s	1516m	1508m	1511sh	1508m	1504m
	1481sh			1500m	–	1496sh
				1496m		
$\delta(CH_2)$	–	1455sh	1468m	1467m	–	1467m
	1436m	1442m	–		1455m	1452m
$\nu_s(COO)$	1390s	–	1411m	1411sh	1411s	1406m
$\omega(CH_2)$	1330sh	1331s	1337sh	1330s	1335sh	1330sh
$\tau(CH_2)$	1323m	1312m				
$\nu_3(NO_3)$	–	1353m	1379m	1391m	–	
		–	1287s	1292s	1302s	1299s
		–			–	
		–				
ν(C–OH)	–	1215s	1232sh	1232sh	–	–
$\varrho(NH_3)$	1124m	1122m	1112m	1113m	1110sh	1112m
	1112sh	–	–	–	1100m	1104m
$\nu_{as}(CCN)$	1041w	1041m	1035m	1037m	1037m	1038m
	–	–	–	–	–	–
$\varrho(CH_2)$	926m	912m	–	–	–	912sh
	911m	–	901m	902sh	908m	904m
ν(CC)	887m	889m	–	–	–	–
	–	866m	864sh	874sh	–	–
$\nu_2(NO_3)$	–	809m	822m	818m	821	819m
	–	–	807sh	–	–	–
$\nu_4(NO_3)$	–	736m	–	727w	–	726vw
δ(COO)	684w	658	702w	697w	691w	694w

Vibration	Gly	$GlyHNO_3$	CrGly	LaCrGly	MnGly	LaMnGly
			–	–		
	–	–			–	671w
δ (COO)				653vw	–	–
	–	–	636vw	634vw	–	–
ω(COO) + (H_2O)	605w	–	601m	600w	–	–
		570m	578sh	582sh	584m	584m
δ(CCO)	499m	497m	510vw	526w	531m	526m
				509m	–	511m
ν(M–O)	–	–	446–430w	455w	(340–290)w	(342–300)w
				428w		
δ(NCC)	356w	303	367sh	–	–	–

s–strong intensity, m–medium intensity, w–weak intensity, sh–shoulder.

Estimation of adiabatic temperature at combustion of fuel–rich glycine–nitrate precursors in air

Reactions for estimation of adiabatic temperature:

$La(NO_3)_3 + Cr(NO_3)_3 + 6C_2H_5NO_2 + 6O_2 = LaCrO_3 + 12CO_2 + 15H_2O + 6N_2$ (1)

$La(NO_3)_3 + Mn(NO_3)_2 + 5C_2H_5NO_2 + 11.5O_2 = LaMnO_3 + 10CO_2 + 12.5H_2O + 5N_2$ (2)

Formula for estimation of adiabatic temperature:

$$T_{ad} = \frac{|\Delta_r H^o_{298}|}{\sum_{i=1}^{m} \nu_i C_{pi} + 3.76 \cdot \nu_{O_2} C_{p_{O_2}}} + 298\,, \quad (1S)$$

where $\Delta_r H_{298}$ is the enthalpy of reaction (2S) or (3S) at 298 K, [J·mol^{-1}]; ν_i are the stoichiometric coefficients of the products; C_{pi} are the heat capacity of the products at constant pressure, [J · mol^{-1} · K^{-1}]. The values of the adiabatic temperature were calculated using the standard values of enthalpy for the initial reagents not taking into account their interaction in the composition of the precursor (Table S2). The dependence of the enthalpy on temperature was not taken into account and the heat capacities of the combustion products were taken at 298 and 800 K. Note, that 800 K was the most often measured temperature in analogous processes of combustion.

Table S3. Enthalpies and heat capacities data used to estimate the adiabatic temperature.

**Compound**	**$\Delta_r H^\circ_{298}$ (kJ·mol^{-1})**	**$C_{p\ 800K}$, (J·mol^{-1}·K^{-1})**	**$C_{p\ 298K}$, (J·mol^{-1}·K^{-1})**
$La(NO_3)_3$ (solid)	–1254.57[1]	–	–
$Mn(NO_3)_2$ (solid)	–575[2]	–	–
$Cr(NO_3)_3$ (solid)	–450[2]	–	–
$C_2H_5NO_2$ (solid)	–524.67[3]	–	–
$LaMnO_3$ (solid)	–1437.99[2]	136.76[2]	102.65[2]
$LaCrO_3$ (solid)	–1534.4[4]	132.82[4]	107.9[4]
H_2O (gas)	–241.81[3]	36.02[3]	33.61[3]
CO_2 (gas)	–393.51[3]	45.52[3]	37.11[3]
O_2 (gas)	0[3]	31.74[3]	29.37[3]
N_2 (gas)	0[3]	30.22[3]	29.12[3]

1. Dean, J.A. *Lange's Handbook of Chemistry*. Fifteenth Ed.; McGRAW–HILL: New York, NY, USA, **1999**, *8*.
2. Jacob, K.T., Attaluri, M. Refinement of thermodynamic data for $LaMnO_3$. *J. Mater.Chem.* **2003**, *13*, 934–942.
3. Kratkii spravochnik fiziko-khimicheskikh velichin (The quick–reference book of physicochemical quantities.) K.P. Mishchenko and A.A. Ravdel′ (Eds.); Leningrad: Khimiya, 1974 (in Russian). https://scholar.google.com/scholar_lookup?title=Kratkii%20spravochnik%20fiziko-khimicheskikh%20velichin &publication_year=1974
4. Bonet, A.; Travitzky, N.; Greil, P. Synthesis of $LaCrO_3$ and $La_{0.9}Ca_{0.1}CrO_3$ by modified glycine nitrate process. *J. Ceram. Sci. Tech.* **2014**, *5*, 93–100.

Table S4. Estimated thermodynamic data for combustion of precursors.

**Precursor**	**Molar ratio of Gly/NO_3**	**φ**	**Gas moles to 1 mole of perovskite**	**$\Delta_r H_{298}$ ($J \cdot mol^{-1}$)**	**T_{ad298} (K)**	**T_{ad800} (K)**
La–Cr–Gly (1)	1	1.8	33	–5031	2953	2675
La–Mn–Gly (2)	1	1.8	27.5	–3943	2005	1835
